# Higher Sensitivity of Foxp3^+^ Treg Compared to Foxp3^-^ Conventional T Cells to TCR-Independent Signals for CD69 Induction

**DOI:** 10.1371/journal.pone.0137393

**Published:** 2015-09-09

**Authors:** Anna Bremser, Maria Brack, Ana Izcue

**Affiliations:** 1 Max-Planck-Institute of Immunobiology and Epigenetics, Freiburg, Germany; 2 Centre for Chronic Immunodeficiency (CCI), University Medical Center Freiburg, University of Freiburg, Freiburg, Germany; 3 Faculty of Biology, University of Freiburg, Freiburg, Germany; Institut Pasteur, FRANCE

## Abstract

T lymphocytes elicit specific responses after recognizing cognate antigen. However, antigen-experienced T cells can also respond to non-cognate stimuli, such as cytokines. CD4^+^ Foxp3^+^ regulatory T cells (Treg) exhibit an antigen-experienced-like phenotype. Treg can regulate T cell responses in an antigen-specific or bystander way, and it is still unclear as to which extent they rely on T cell receptor (TCR) signals. The study of the antigen response of Treg has been hampered by the lack of downstream readouts for TCR stimuli. Here we assess the effects of TCR signals on the expression of a classical marker of early T cell activation, CD69. Although it can be induced following cytokine exposure, CD69 is commonly used as a readout for antigen response on T cells. We established that upon in vitro TCR stimulation CD69 induction on Foxp3^+^ Treg cells was more dependent on signaling via soluble factors than on TCR activation. By contrast, expression of the activation marker Nur77 was only induced after TCR stimulation. Our data suggest that Treg are more sensitive to TCR-independent signals than Foxp3^-^ cells, which could contribute to their bystander activity.

## Introduction

Foxp3-expressing regulatory T cells (Treg) are essential for establishing tolerance [1]. In general, T cells are activated and maintained through TCR signals. While Treg can survive without TCR, they require TCR signals to become activated and to be able to fully mediate their suppressive function [2, 3]. TCR signals are also necessary to suppress the activation of effector T cells with a different specificity in vitro (bystander suppression) [4, 5]. While several reports indicate that cognate antigen is required in vivo for Treg division and persistence under competitive settings [6, 7], it remains unclear whether Treg act in vivo in an antigen-specific manner or inhibit effector cells via bystander activity [8].

One of the reasons for this uncertainty is the lack of an assay to quantitate Treg specificity. So far, in vivo studies on Treg specificity have mostly been performed on TCR transgenic mice [9] or using tetramers, allowing identification of specificities to only one epitope [10]. Although Treg do not readily proliferate in vitro [4, 11, 12], the degree of proliferation of Treg in response to antigen-pulsed dendritic cells has been used to quantify Treg reactivity in certain settings [13, 14]. Other approaches, such as organ-specific regulation assays in vivo [6, 7] or TCR cloning and identification of specificity [15, 16] are very time-consuming and the outcome can be obscured by factors such as bias during cloning.

In order to identify earlier readouts that may allow a more direct assessment of antigen specificity, we tested the suitability of the early activation markers CD69 and Nur77 to assess Treg response to TCR signals in vitro. CD69 has long been used as a T cell activation marker, but it can be induced by stimuli other than TCR ligation, such as type I interferon, so that its application is limited in conditions of inflammation [17–19]. Nur77, encoded by *Nr4a1*, was identified some time ago as an early response gene in T cells [20]. A recently generated *Nr4a1*-based GFP reporter showed that *Nr4a1*-GFP expression can be used to estimate TCR affinity, and it does not appear to be induced on CD8^+^ T cells under inflammatory conditions [18]. One caveat regarding the use of this reporter is the stability of the GFP. In *Nr4a1*-GFP reporter mice, Treg exhibit high ex vivo levels of GFP, probably arising from continuous antigenic stimulation in the periphery [18]. It is possible that the high basal GFP masks GFP induced by antigen recognition in an ex vivo assay.

Here we analyzed the response of Treg to TCR stimulation in vitro using two markers of early activation, CD69 and Nur77, as a readout. Both were readily induced on Treg upon TCR activation in vitro and, as described for Foxp3^-^ T cells, the expression of CD69 was more sustained than that of Nur77. However, in the presence of antigen-presenting cells (APCs), the expression of CD69 in Treg was mostly induced by soluble factors rather than antigen-dependent signals. We further demonstrate that Foxp3^+^ T cells are more sensitive to cytokine-mediated induction of CD69 than their Foxp3^-^ counterparts. In contrast, Nur77 induction was specific for TCR signals. Hence, Treg appear to rely more on soluble signals than other CD4^+^ T cell subsets. This suggests that some CD4^+^ T cell activities that are normally directed by antigen in non-Treg may be induced by bystander activation in Foxp3^+^ cells.

## Results

### CD69 is induced on Foxp3^+^ T cells in antigen-dependent and-independent ways

**Fig 1:**
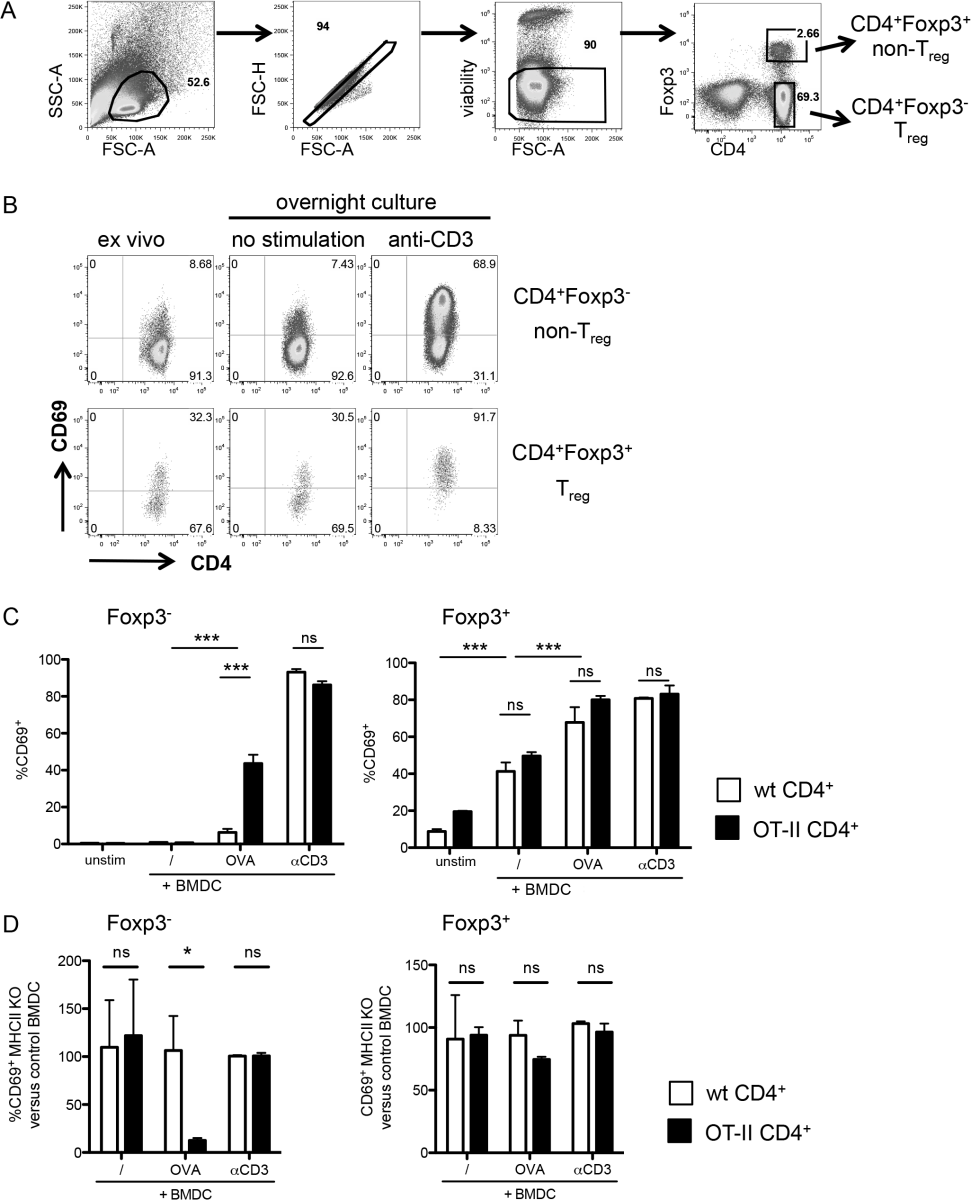
Foxp3^+^ cells upregulate CD69 after TCR activation in an antigen-non-specific manner. (A) Gating strategy on CD4^+^ enriched T cells after overnight culture without stimulation. Cells are gated in the lymphocyte gate based on forward versus side scatter, then doublets are excluded based on forward scatter height versus amplitude and live cells are gated as negative for the dead cell marker. Finally Foxp3^+^ and Foxp3^-^ CD4^+^ T cells are gated using CD4 and Foxp3 expression. This gating strategy was applied throughout the analyses unless otherwise stated. (B) CD69 expression on Foxp3^-^ (top row) and Foxp3^+^ (bottom row) CD4^+^ cells before and after overnight stimulation with or without anti-CD3 stimulation. Staining is representative of at least three independent experiments. (C) CD69 expression on Foxp3^-^ (left) and Foxp3^+^ (right) enriched CD4^+^ T cells from either wild-type (white bars) or OT-II (black bars) donor mice, after overnight culture with BMDCs. Unstim, control without BMDC; /, unpulsed BMDC; OVA, OVA-pulsed BMDC, anti-CD3, anti-CD3-coated BMDC. Data are representative of four independent experiments with each 3 replicates per group. (D) Normalized MHCII KO BMDC-induced CD69 expression compared to control BMDC-induced CD69. The graphs show the frequency of CD69^+^ cells among CD4^+^ Foxp3^-^ (left) and Foxp3^+^ (right) T cells after stimulation with MHCII KO BMDC, normalized to CD4^+^ Foxp3^-^ and Foxp3^+^ T cells stimulated with wild type BMDC for each given condition. 100% represents the same CD69 expression by cells stimulated with control or MHCII KO BMDC. Data show mean + SD, *p ≤ 0.05; **p ≤ 0.01; *** p ≤ 0.001, ns, non significant. Data are representative of three independent experiments with each n = 3 per group.

CD69 has long been used as a marker for T cell activation, and also as a marker for antigen-activated Treg [21–23]. Indeed, in vivo studies suggest that CD69 is increased on Treg upon activation, although it has not been assessed if this occurred in an antigen-specific way. To test if CD69 expression can be used as a readout for Treg activation, we checked the expression of CD69 on Treg after in vitro stimulation. Ex vivo Treg present higher levels of CD69 than Foxp3^-^ cells [24]. To reduce this effect, we initially pretreated the cells with unlabeled anti-CD69 antibody before culture. We then stimulated bulk CD4^+^ T cells with plate-bound anti-CD3 and assessed the expression of CD69 after overnight incubation (Fig 1A and 1B). IL-2 was added to all cultures to enhance Treg survival. Addition of IL-2 did not affect the levels of CD69 (data not shown). Overnight culture in the absence of exogenous stimuli did not influence CD69 expression, but TCR stimulation increased CD69 on both Foxp3^-^ and Foxp3^+^ cells (Fig 1B). Although the mean fluorescence levels of CD69 were slightly lower in Treg, in contrast to the findings in a previous report [25] most Foxp3^+^ cells expressed CD69 after TCR stimulation.

We then employed a system using APCs to test the antigen-specificity of CD69 upregulation. Bone marrow-derived dendritic cells (BMDC) were incubated with ovalbumin (OVA) and co-cultured overnight with either polyclonal control CD4^+^ T cells or OT-II CD4^+^ T cells bearing a transgenic T cell receptor specific for OVA peptide 323–339 [26]. As expected, CD69 expression on Foxp3^-^ cells was only greatly induced when TCR transgenic—but not wild-type—T cells were cultured with BMDC that had been pulsed with OVA (Fig 1C). Stimulation of OT-II Foxp3^-^ T cells with BMDC in the absence of OVA, or of non transgenic CD4^+^ T cells with OVA-pulsed BMDC did not result in robust CD69 induction.

As a control, both polyclonal and TCR-transgenic Foxp3^-^ CD4^+^ T cells increased CD69 to similar levels when stimulated with anti-CD3. The higher degree of CD69 induction after anti-CD3 compared to OVA stimulation may reflect a less efficient antigen presentation for OVA.

In contrast, Foxp3^+^ T cells upregulated CD69 when cultured with BMDC irrespective of the presence or absence of OVA (Fig 1C). While there was a higher induction of CD69 by OVA-pulsed BMDC, this increase was not antigen-specific as it was observed on both TCR transgenic and non-transgenic Foxp3^+^ T cells (Fig 1C). It must be borne in mind that Treg developing in RAG-sufficient TCR transgenic mice are more likely to bear non-transgenic TCRs than Foxp3^-^ cells. It therefore remained possible that they were activated by endogenous antigens presented by the BMDCs. Since most Treg recognize antigens in the context of MHCII [27], we assessed whether the stimulatory capacity of BMDC was dependent on MHCII (Fig 1D). The levels of CD69 expression on unstimulated T cells or T cells stimulated with anti-CD3 were unaffected by the lack of MHCII on BMDC, as expected. Similarly, CD69 induction on conventional Foxp3^-^ TCR-transgenic cells incubated with OVA-pulsed BMDC was dependent on MHCII (Fig 1D). In contrast, MHCII-deficient and control BMDC induced similar levels of CD69 on Foxp3^+^ Treg. All in all, these data suggest that, unlike conventional naive CD4^+^ Foxp3^-^ T cells, Treg respond to BMDC stimulation in an antigen-non specific manner.

### Enhanced CD69 response of Foxp3^+^ cells to cytokines

**Fig 2:**
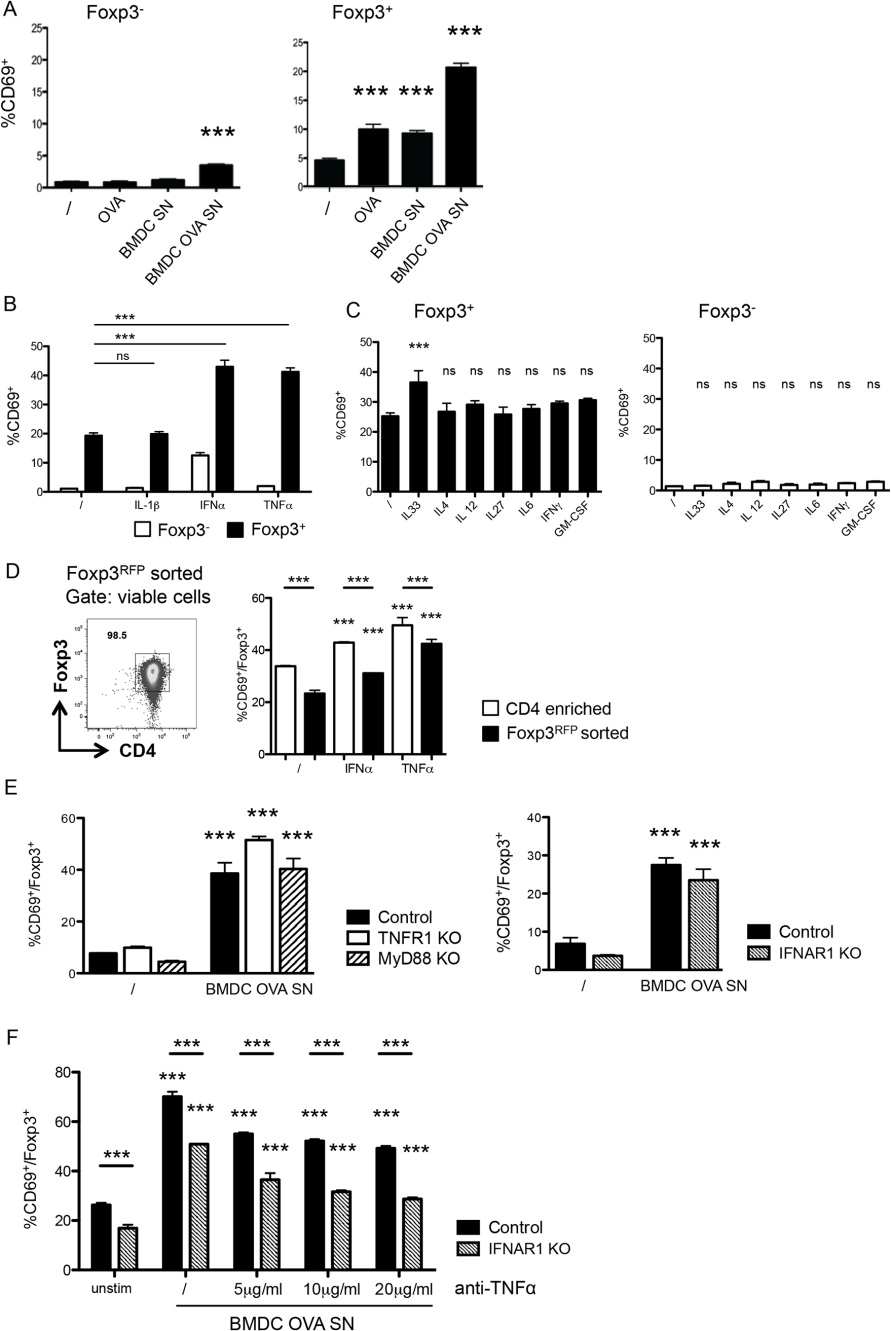
Foxp3^+^ cells preferentially upregulate CD69 in response to soluble factors. (A) CD69 expression on Foxp3^-^ (left) and Foxp3^+^ (right) enriched CD4^+^ T cells after overnight incubation with or without BMDC-conditioned culture medium. /, control medium; OVA, medium supplemented with OVA; BMDC SN, supernatant from BMDCs cultured overnight with fresh medium; BMDC OVA SN, supernatant from BMDCs cultured overnight with medium supplemented with OVA. Data are representative of at least two independent experiments. (B) CD69 expression on Foxp3^-^ (white bars) and Foxp3^+^ (black bars) among enriched CD4^+^ T cells cultured overnight without additional cytokines (/) or with IL-1β, IFN-α or TNF-α. Data are representative of at least two independent experiments. (C) CD69 expression on Foxp3^+^ among enriched CD4^+^ T cells cultured overnight without additional cytokines (/) or with IL-33, IL-4, IL-12, IL-27, IL-6, IFN-γ, or GM-CSF. (D) Representative FACS plot and response to stimulation of sorted CD4^+^ Foxp3^RFP+^ cells. Right: representative FACS plot showing the purity of Foxp3^RFP+^ cells after sort. Cells are gated on forward and side scatter and doublets and dead cells are excluded as in Fig 1A. Plot shows CD4 versus intracellular Foxp3 staining. Left, CD69 expression on Foxp3^+^ among enriched CD4^+^ T cells (white bars) or Foxp3^RFP+^ sorted (black bars) CD4^+^ T cells, cultured overnight without additional cytokines (/) or with IFN-α or TNF-α (left). (E) CD69 expression on Foxp3^+^ among enriched CD4^+^ splenocytes from TNFR1 KO, Myd88 KO and ctrl C57BL/6 or from IFNAR1 KO and control 129 mice, cultured overnight with control medium (/) or with supernatant from OVA stimulated BMDCs (BMDC OVA SN). (F) CD69 expression on Foxp3^+^ among enriched CD4^+^ T cells of IFNAR1 *KO* and control mice cultured overnight with control medium (unstim) or with supernatant from OVA stimulated BMDCs (BMDC OVA SN) with or without addition of different concentrations of blocking anti-TNF-α antibody. Data are representative from two independent experiments. Data show mean + SD, *** p ≤ 0.001 compared to unstimulated control, unless comparison indicated by line below the stars, n ≥ 3 per group.

We proceeded to further characterize the factors inducing CD69 on Foxp3^+^ T cells in an antigen non-specific manner after culture with OVA-pulsed BMDC. We therefore checked if incubation of BMDC with the OVA protein could induce the production of soluble mediators causing CD69 upregulation. Indeed, induction of CD69 on Foxp3^+^ T cells could already be observed after addition of OVA without BMDC supernatant or after addition of the supernatant of non-pulsed BMDC (Fig 2A). The effect was strongest when supernatant from OVA-pulsed BMDC was added. Hence, soluble factors can account at least partly for the observed antigen-non specific induction of CD69 on Treg. In contrast, adding either OVA or BMDC supernatant did not induce any increase in CD69 on conventional Foxp3^-^ T cells; and OVA-pulsed BMDC supernatant only promoted minor CD69 expression on Foxp3^-^ T cells.

Hence, certain factors in the OVA solution and BMDC-derived soluble factors can induce CD69 on Treg. Commercial OVA is known to be contaminated with LPS, which can promote cytokine production by BMDC [28]. Several cytokines have been reported to mediate CD69 upregulation in vivo [17, 19]. Both IFN-α and TNF-α, known CD69 inducers [17, 29], promoted CD69 induction in a substantial fraction of Treg (Fig 2B). In contrast, culture with IL-1β, which shares some signaling components with TNF-α [30], did not affect CD69 expression on Treg. Foxp3^-^ T cells exhibited a much weaker CD69 response to the cytokines tested. In conventional CD4^+^ Foxp3^-^ T cells, IFN-α, the cytokine with the strongest effect, induced CD69 on about 10% of all Foxp3^-^ T cells, which compared to CD69 expression on 40% of Foxp3^+^ T cells after stimulation with IFN-α or TNF-α (Fig 2B).

This observation suggested that Treg can potentially respond to other homeostatic/inflammatory cytokines. We found that IL-33, which is recognized by a subset of Treg [31], induced CD69, although to a lower degree than IFN-α or TNF-α (Fig 2C). In contrast, other tested cytokines (IL-4, IL-12, IL-27, IL-6, IFN-,γ, GM-CSF) did not increase the expression of CD69 (Fig 2C). We confirmed the induction of CD69 in response to IFN-α and TNF-α in cultures with sorted Treg, identified through a *Foxp3*-RFP reporter (Fig 2D). However, we consistently observed a reduced response of Foxp3^+^ T cells in sorted compared to enriched samples pointing to additional indirect effects for the induction of CD69 acting on Foxp3^+^ T cells.

We then addressed whether one of these cytokines is responsible for the CD69 response of Treg to the BMDC supernatant. To this aim, we used CD4^+^ T cells isolated from mice deficient for the IFN-αβ receptor IFNAR1, for the TNF type 1 receptor—considered the main receptor for soluble TNF-α [32]—or for the adaptor protein MyD88, which mediates responses to IL-1, LPS and other stimuli. We established that Foxp3^+^ cells from these mice still responded to BMDC supernatant by upregulating CD69 (Fig 2E). The simultaneous absence of both TNF-α and IFN-α signals reduced, but did not completely abolish, the induction of CD69 in response to BMDC supernatant (Fig 2F). This result suggests that Treg can react to multiple factors present in the supernatant. Together, our data show that, compared to Foxp3^-^ cells, Treg display an enhanced tendency to respond to cytokine stimulation by CD69 induction, and that CD69 expression on Treg ex vivo is unlikely to reflect antigenic stimulation alone.

### Nur77 is induced on Treg in an antigen-dependent way

**Fig 3:**
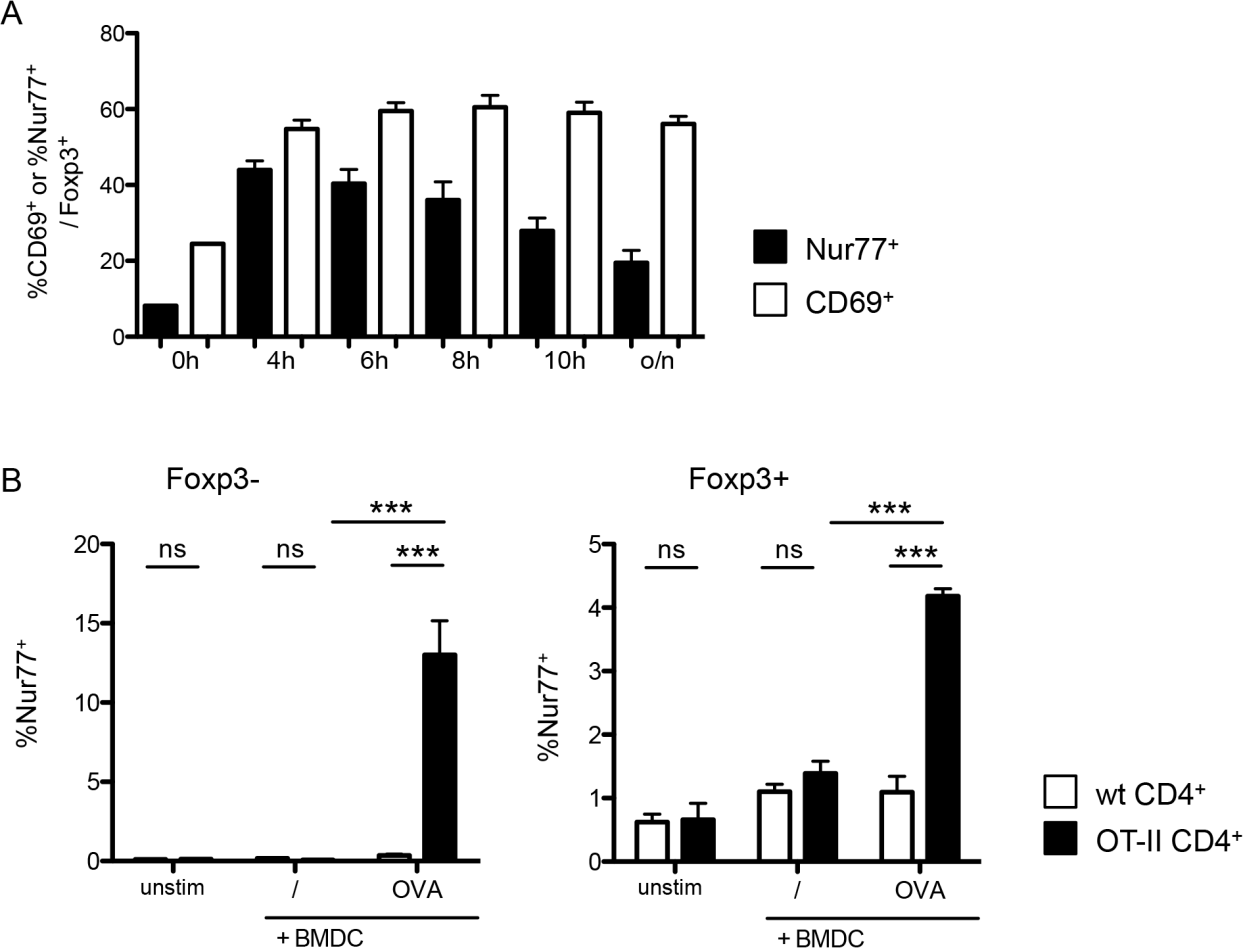
Foxp3^+^ cells upregulate Nur77 after TCR activation in an antigen-specific manner. (A) Time course of Nur77 (black bars) and CD69 expression (white bars) on Foxp3^+^ T-cells among sorted CD25^+^ CD4^+^ splenocytes stimulated with plate-bound αCD3 and 1U/ml IL-2. Data are representative of 3 independent experiments with n ≥ 3. (B) Nur77 expression on Foxp3^-^ (left) and Foxp3^+^ (right) among enriched CD4^+^ T-cells from either wild-type (white bars) or OT-II (black bars) mice, co-cultured overnight with unstimulated, OVA pulsed or αCD3 coated BMDCs. Data are representative of two independent experiments, with each n = 3 per group. unstim, control without BMDC; /, unpulsed BMDC; OVA, OVA-pulsed BMDC, anti-CD3, anti-CD3-coated BMDC. Data show mean + SD, *p ≤ 0.05, *** p ≤ 0.001; ns, non significant. Data are representative of four independent experiments each with n = 3 per group.

In contrast to CD69, Nur77 induction has been reported to be antigen-specific and independent of inflammation [18]. We then tested the specificity of Nur77 as a Treg cell TCR activation marker, measuring endogenous Nur77 expression by intracellular staining to determine T cell activation. In contrast to CD69, Nur77 induction as a result of TCR engagement is transient [18]. Kinetic analysis demonstrated that Nur77 expression in Foxp3^+^ T cells is induced within hours after stimulation, but is not maintained and is greatly reduced after overnight incubation compared to earlier time points (Fig 3A). Indeed, when we tested Nur77 induction in response to anti-CD3 stimulation, we found that, after overnight stimulation, the frequency of Nur77^+^ cells was much lower than that of CD69^+^ cells, probably due to the transient nature of endogenous Nur77 expression (Fig 3A). However, and in contrast to CD69, Nur77 expression was antigen-specific for both Foxp3^+^ and Foxp3^-^ T cells (Fig 3B), as only TCR-transgenic T cells responded to OVA-pulsed BMDC.

**Fig 4:**
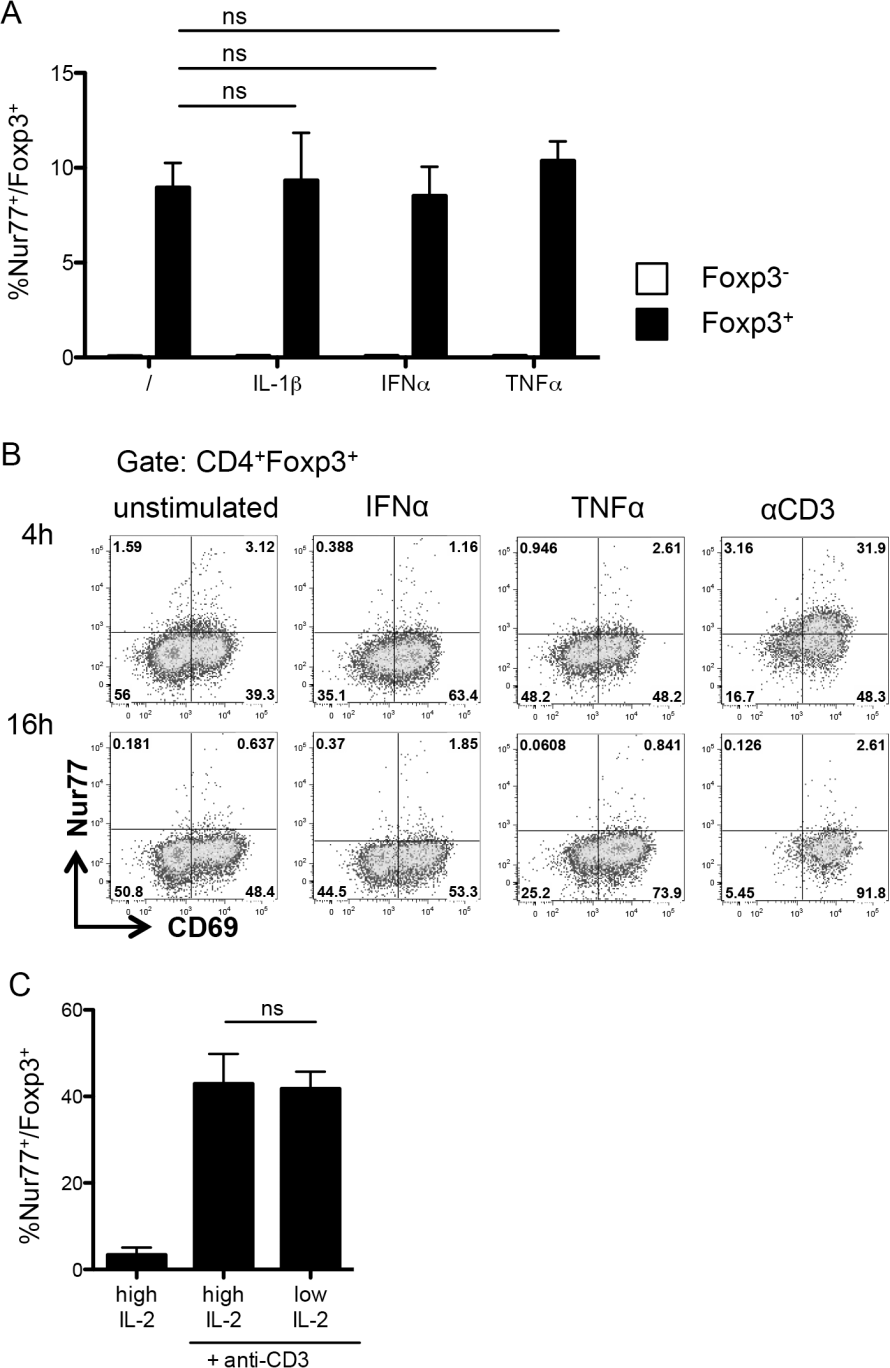
Nur77 intensity is not modulated by cytokines. (A) Nur77 expression on Foxp3^-^ (white bars) and Foxp3^+^ (black bars) CD4^+^ T cells from the culture in Fig 2B. Data are representative of at least two independent experiments. (B) Representative plots of Nur77 versus CD69 expression on Foxp3^+^ among enriched CD4^+^ T cells, stimulated for 4 hours (top row) or 16 hours (bottom row) without additional cytokines (/), with IFN-α, TNF-α, or plate-bound αCD3. Data are representative of two independent experiments. (C) Nur77 expression on Foxp3^+^ cells among sorted CD25^+^ CD4^+^ splenocytes stimulated with plate-bound αCD3 with low (1U/ml) or high (1000U/ml) IL-2 for 6 hours. Data are representative of four independent experiments. Data show mean + SD, ns, non significant.

We further checked if Nur77 expression was affected by cytokine stimulation. Unlike CD69, addition of IL-1-β, TNF-α, or IFN-α did not affect Nur77 expression after overnight culture (Fig 4A) nor after 4h (Fig 4B). Since we performed our cultures in the presence of IL-2, we proceeded to check if the dose of IL-2 would influence Nur77 induction. As was the case for the tested cytokines, the dose of IL-2 did not modify the frequency of Nur77-expressing cells (Fig 4C). Hence, Nur77 appears to be a reliable reporter of TCR stimulation in Treg.

**Fig 5:**
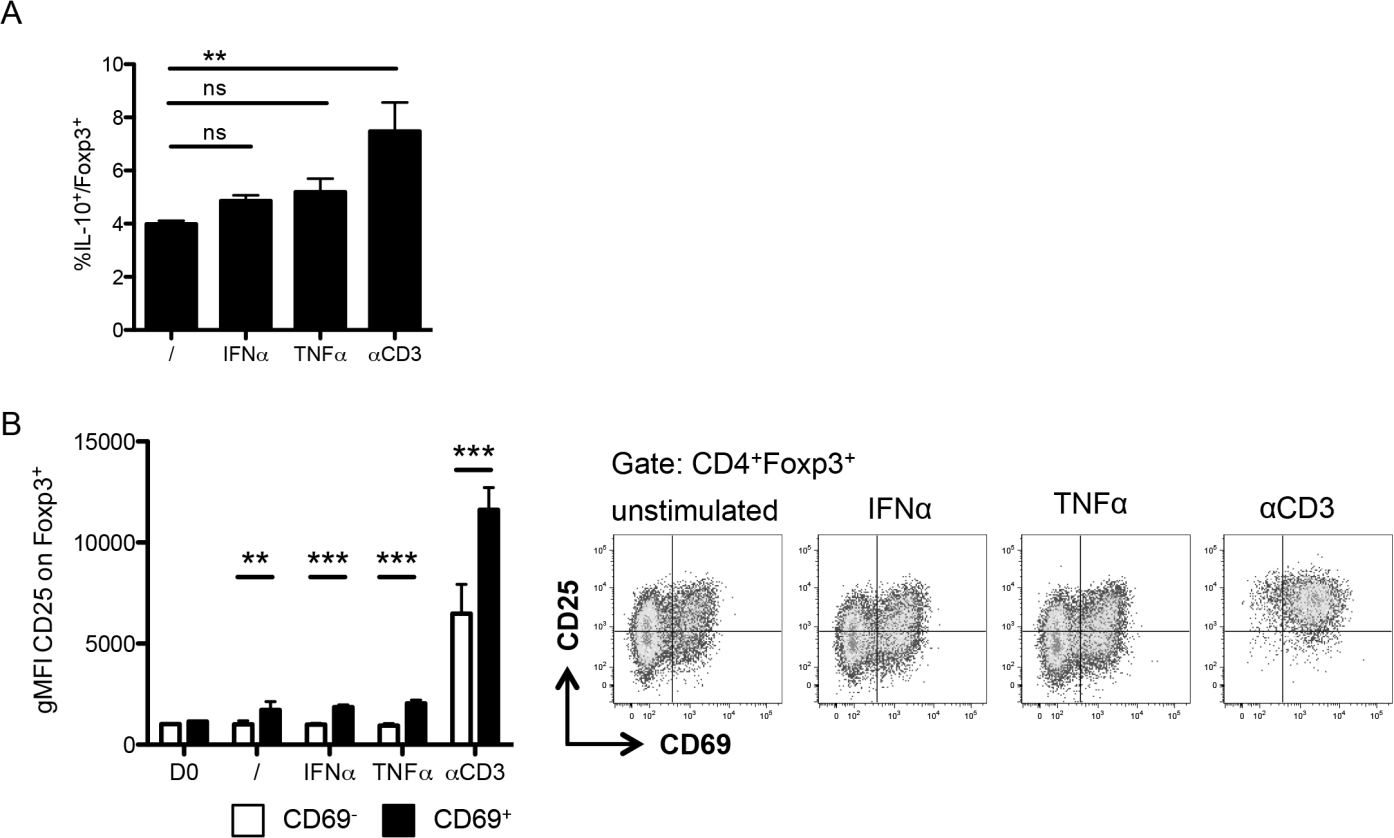
IL-10 and CD25 expression by cytokine-activated CD69^+^ Treg. (A) Frequency of IL-10^+^ cells by IL-10 intracellular staining on Foxp3^+^ among enriched CD4^+^ T cells stimulated overnight without additional cytokines (/), with IFN-α, TNF-α, or plate-bound αCD3. (B) Expression of CD25 by geometric mean fluorescence on CD69^+^ (black bars) and CD69^-^ (white bars) (left) and representative plots (right) of Foxp3^+^ among enriched CD4^+^ T cells stimulated as in (A). Data show mean + SD, **p ≤ 0.01, *** p ≤ 0.001; ns, non significant. Data are representative of two independent experiments.

These data raise the question whether TCR-independent responses affect Treg suppressive function. This aspect is difficult to analyze, since functional regulation studies typically rely on the inhibition of activated T cells, which secrete cytokines and can therefore activate Treg in an antigen-nonspecific way. We therefore assessed the expression of the immunoregulatory cytokine IL-10 and the IL-2 receptor CD25, which participates in immunosuppression through IL-2-deprivation. In contrast to anti-CD3 stimulation, cytokine activation affected neither the production of IL-10 nor the expression of CD25 on Treg (Fig 5A & 5B). CD69^+^ Foxp3^+^ cells expressed higher levels of CD25 than CD69^-^ Foxp3^+^ cells across all conditions, while PMA stimulation to assess IL-10 expression induced CD69 expression on all T cells, precluding separate analysis of CD69^+^ and CD69^-^ cells (Fig 5B and data not shown).

Hence, although cytokines promote the expression of CD69 and may affect the migratory pattern of Treg, antigen-specific activation seems responsible for the induction of suppressor molecules.

## Discussion

Although TCR signaling and antigen specificity are bound to play a role in Treg homeostasis and function, the exact role of antigen recognition in Treg is still unclear. Here we tested the suitability of CD69 and Nur77 as markers to measure Treg response to TCR signals. We established that, CD69 was strongly induced on Foxp3^+^ T cells—in contrast to Foxp3^-^ T cells—in an antigen-independent way in vitro, whereas transient Nur77 expression was TCR-specific.

CD69 is expressed on all T cells upon TCR activation. However, it is not specific for TCR signals, as it can also be induced by cytokines such as IFN-α [17, 19]. Despite this caveat, CD69 is still widely used as a marker to estimate the frequency of antigen-responding T cells. Our results demonstrate that, while this may be a reasonable approach for the population of conventional Foxp3^-^ T cells, it can be highly misleading when applied to Foxp3^+^ Treg, as many of these will increase CD69 expression in the absence of their cognate antigen. We have established that at least part of this effect is due to soluble factors, and that it can be mediated not only by IFN-α, but also by other cytokines such as TNF-α, which plays an important role in Treg homeostasis [33]. Although we show that both TNF-α and IFN-α suffice to induce CD69 on Treg, neither type I interferon receptor nor the TNF-α receptor TNFR1 was essential for the supernatant-mediated CD69 expression. Blockade of TNF-α with an antibody reduced, but did not abolish, CD69 induction, even in the absence of type I interferon signals. Hence, our data suggest that several cytokines can redundantly induce CD69 expression on Treg. This flexibility fits with a report showing that many different cytokine combinations can induce CD69 on effector CD8^+^ T cells in the absence of TCR signals [19]. As Treg displayed antigen non-specific induction of CD69 in response to several cytokines, it is likely that CD69 expression on Treg ex vivo does not merely reflect recent TCR activation.

We have not explored the reasons for the differing cytokine sensitivity of Treg compared to conventional CD4^+^ Foxp3^-^ T cells. However, Treg have been described to possess an effector-like phenotype and high levels of cytokine receptors which could play a role in Treg function, allowing them to compete with effector cells for growth factors [34]. Higher receptor levels could also account for the enhanced Treg sensitivity to cytokines. Their readiness to upregulate CD69 could also enhance Treg retention in lymphoid organs during the establishment of an inflammatory response, allowing them to scan an inflammatory environment to limit misdirected or excessive inflammation. Beyond its role in migration [17], CD69 has also been reported to play a role in CD4^+^ T cell and Treg function [35–37]. As S1P1 has been reported to reduce Treg activity [38], the expression of CD69, which inhibits S1P1 function and reduces its surface expression [17, 39], might be able to promote Treg activity in an antigen non-specific way. Indeed, some reports indicate that CD69 could play a role in Treg function [40], while CD69-deficient Treg have a reduced function [24]. CD69 has been reported to also have S1P1-independent effects, so that it could potentially influence Treg in multiple ways [35, 36].

Nur77 induction has been reported to reflect TCR-mediated activation better than CD69 [18]. Our results also confirm these data for peripheral Treg. A reporter mouse strain with GFP under the control of a sequence from the *Nr4a1* promoter has recently been used to track Treg responses to antigens in the thymus [18, 41]. The results are coherent with the notion that Treg recognize self-antigens with higher affinity than conventional T cells. Peripheral Treg ex vivo were GFP positive, which indicates that they probably undergo sustained TCR stimulation [18]. However, in vitro, the basal Treg GFP expression may mask differences in GFP induction in response to antigen. Nur77 can also be detected with specific antibodies, allowing direct measurement of Nur77 protein. Due to the transient nature of Nur77 expression after TCR activatioin, direct Nur77 staining only reflects the immediate Nur77 induction and thus avoids the pitfall of previous Nur77 expression. However, the rapid disappearance of Nur77 makes it difficult to estimate the number of cells responding to a given antigen, since only a certain fraction of responding T cells will express Nur77 at a given time point. Nur77 expression can still be a useful tool for monitoring modulation of TCR responses in Treg, rather than for comparing the response of Foxp3^+^ T cells to different antigens.

In summary, our present results clearly show that while CD69 is a reasonable marker for TCR activation in conventional T cells, this is not the case for Treg. Caution is required when interpreting the significance of higher CD69 expression on Foxp3^+^ cells in different settings. On the other hand, the widespread upregulation of CD69 upon antigen non-specific,inflammatory stimuli may promote retention of Treg in the lymphoid organs during the immune response, thus increasing immunosurveillance. More detailed analysis of the kinetics of Treg recirculation may help us understand the dynamics of immune regulation during response to infection and autoimmune responses.

## Material and Methods

### Mouse strains

Wild-type C57BL/6, Foxp3-IRES-mRFP [42] MHCII KO [43] and OT-II [44] mice were kept and bred under SPF conditions at the animal facility of the Max-Planck Institute of Immunobiology and Epigenetics. TNFR1 KO [45] and MyD88 KO [25] on a C57BL/6 background, and IFNAR1 KO [46] on a 129 background were kindly provided by Marina Freudenberg and P. Stäheli. All experiments were approved by the institutional review board and the local government (Regierungspraӓsidium Freiburg, Referat 35).

### T cell stimulation

CD4^+^ T cells from spleens were enriched using a CD4^+^ isolation kit (Dynabeads Untouched Mouse CD4 Cells, Life Technologies). Where indicated, CD4^+^CD25^+^ T cells or CD4^+^Foxp3^RFP+^ were sorted from CD4^+^-enriched splenocytes on a cell sorter BD Aria II or III (BD).

Flat-bottom 96-well plates were pre-coated with 1 μg or 5 μg purified anti- mouse CD3ε (145-2C11, Biolegend) at 37°C for a minimum of 30 minutes. Cells were seeded at a density of 10^6^ cells/well (CD4^+^-enriched cells) or at 10^5^ cells/ well (FACS-sorted CD4^+^CD25^+^ cells) in RPMI containing 10% fetal calf serum and 1000U recombinant human IL-2 (cat# 200–02, PeproTech) unless otherwise stated. For some CD69 stimulations, cells were blocked prior to culture with 5μg/ml anti-mouse CD69 (H1.2F3, eBioscience) for 15 minutes at 37°C, and the culture medium was additionally supplemented with 50 μM SEW2871 (cat# CAS 256414-75-2, Cayman Chemical). Where indicated, culture medium was supplemented with 5,000U/ml recombinant murine IL-1β (cat# 211-11B, Peprotech), 1,000U/ml recombinant murine TNF-α (cat# 315-01A, Peprotech), 1,000U/ml recombinant murine IFN-α (cat# 407293, Calbiochem), 20ng/ml recombinant murine IL-4 (cat# AF-214-14, Peprotech), 50ng/ml recombinant murine IL-6 (cat# 575706, Biolegend), 20ng/ml recombinant murine IL-12 (p70) (cat# 577002, Biolegend), 100ng/ml recombinant murine IL-27 (cat# 12340273, Immunotools), 2ng/ml recombinant murine IL-33 (cat# 580502, Biolegend), 100ng/ml recombinant murine IFNγ (cat# 575306, Biolegend), 10ng/ml recombinant murine GM-CSF (cat# 315–03, Peprotech). After the indicated time of incubation at 37°C, cells were harvested and analysed by flow cytometry.

### BMDC co-culture

Bone marrow from mouse femurs was harvested and cultured for 7 days in RPMI containing 10% fetal calf serum, 20 mM HEPES (PAA) and 20 ng/ml recombinant murine GM-CSF (cat# 315–03, Peprotech). BMDCs were pulsed overnight in flat-bottom 96-well plates with a seeding density of 3x10^5^ cells/well in RPMI containing 10% fetal calf serum with or without the presence of 100 μg/ml Ovalbumin, Grade V (cat# A5503, Sigma). Supernatant of BMDC after overnight culture was collected and cells washed. CD4^+^ T cells were prepared as described above and seeded onto pulsed BMDCs at a density of 10^6^ cells/well and cultured overnight in RPMI containing 10% fetal calf serum. The medium was supplemented as described above.

### Antibodies and Flow cytometry

Single cell suspensions were stained in 96-well plates (10^6^ cells per well). The following conjugated antibodies were purchased from eBioscience: TCR-β (H57-597), CD4 (GK 1.5), CD69 (H1.2F3), CD25 (PC61.5). Intracellular staining was performed with the eBioscience permeabilization and fixation kit using the following antibodies from eBioscience: Foxp3 (FJK-16s), Nur77 (12.14). For intracellular IL-10 (JES5-16E3, Biolegend) staining, cells were stimulated in RPMI containing 10% fetal calf serum with 0.1μg/ml Phorbol 12-myristate 13 acetate (cat# PE-160, Enzo), 1μg/ml Ionomycin-Ca-salt (cat#26379, Serva) and Monensin (cat# 420701, Biolegend) for 4 hours at 37°C before staining. Dead cells were excluded by staining with Fixable Viability Dye (eBioscience). All flow cytometry experiments were acquired using a BD LSR II cytometer or LSRFortessa (BD). Flow Jo Version 8.8.7 was used for data analysis.

### Statistical analysis

Statistical analysis was performed using GraphPad Prism applying a 2-tailed unpaired Student’s t test when only two groups were tested, or ANOVA with Bonferroni-Post-test where more than two groups were tested. Differences were considered statistically significant when p < 0.05.
